# The role of white matter in the symptomatology of dissociation in patients with borderline personality disorder

**DOI:** 10.1192/j.eurpsy.2025.1927

**Published:** 2025-08-26

**Authors:** P. Podwalski, B. Dawidowski, L. Franczak, L. Zwarzany, W. Poncyliusz, J. Samochowiec

**Affiliations:** 1 Department of Psychiatry; 2 Diagnostic Imaging and Interventional Radiology Unit, Pomeranian Medical University, Szczecin, Poland

## Abstract

**Introduction:**

Borderline personality disorder (BPD) is characterized by instability in the area of one’s own self and affective reactions, instability in relationships, and impulsive behavior. BPD patients experience dissociative or quasi-psychotic symptoms much more often than the general population. These symptoms directly affect the daily functioning of patients, often preventing them from taking up professional work or having a stable family life. The etiology of dissociative experiences in BPD patients is still unknown. One of the biological models suggests that biological changes in the brain occur on the basis of a traumatic experience, which can produce symptoms. The arcuate fasciculus (AF) is a structure of white matter that interconnects Broca’s area and Wernicke’s area in the brain. AF is often considered in the context of research on psychotic symptoms. For this reason, we hypothesize that AF may be related to dissociative symptoms in BPD patients.

**Objectives:**

The aim of our study was to investigate a relationship between the integrity of AF and symptomatology of dissociation in patients with borderline personality disorder.

**Methods:**

45 BPD subjects and 43 healthy controls (HC) participated in the study. A DTI analysis was performed on all study participants. The psychopathology of BPD was assessed using the Dissociative Experiences Scale - Taxon (DES-T). The AF analysis was then conducted using fractional anisotropy (FA) parameter.

**Results:**

BPD significantly more often than the control group experienced dissociative experiences. We could not identify differences in the integrity of the AF between the two groups. Nevertheless, we examined the correlation between the quality of the AF structure and the severity of dissociative symptoms (r=-0.0299, p=0.039).

**Conclusions:**

The attenuation of the structure of AF may be involved in symptomatology of dissociation in patients with BPD. Further structural brain studies are needed in the BPD population.

**Disclosure of Interest:**

None Declared

